# Development of Efficient One-Pot Methods for the Synthesis of Luminescent Dyes and Sol–Gel Hybrid Materials

**DOI:** 10.3390/ma15010203

**Published:** 2021-12-28

**Authors:** Maria Zdończyk, Bartłomiej Potaniec, Marcin Skoreński, Joanna Cybińska

**Affiliations:** 1 Faculty of Chemistry, University of Wroclaw, F. Joliot-Curie 14 Street, 50-383 Wrocław, Poland; maria.zdonczyk@chem.uni.wroc.pl; 2 Advanced Materials Synthesis Group, Łukasiewicz Research Network—PORT Polish Center for Technology, Stabłowicka 147 Street, 54-066 Wrocław, Poland; bartlomiej.potaniec@port.lukasiewicz.gov.pl (B.P.); marcin.skorenski@port.lukasiewicz.gov.pl (M.S.)

**Keywords:** luminescence, sol–gel, organic dyes, fluorescein derivative

## Abstract

This paper presents a comparison of the simultaneous preparation of di-*O*-alkylated and ether–ester derivatives of fluorescein using different methods (conventional or microwave heating). Shortening of the reaction time and increased efficiency were observed when using a microwave reactor. Moreover, described here for the first time is the application of a fast, simple, and eco-friendly ball-assisted method to exclusively obtain ether–ester derivatives. We also demonstrate that fluorescein can be effectively functionalized by *O*-alkylation carried out under microwave or ball-milling conditions, saving time and energy and affording the desired products with good yields and minimal byproduct formation. All the synthesized products as well as pH-dependent (prototropic) forms trapped in the SiO_2_ matrix were examined using UV–Vis and fluorescence spectroscopy.

## 1. Introduction

Fluorescein, first synthesized by von Bayer [1], and its derivatives are well-known fluorescent dyes [2,3,4]. In solution, the prototropic forms of fluorescein-like dyes are widely described to exist as an equilibrium between the various forms. The various forms reported in the literature are cationic, neutral form with three structures (quinoid, lactone, and zwitterionic), monoanionic, and dianionic [5,6]. Calculations for the determination of their electronic structure were carried out using computational methods [7]. It has also been reported that colorless forms of lactone and colored quinoid occur in equilibrium in aqueous solutions [8]. Three acid–base equilibrium points for aqueous solutions are known to occur at 2.1, 4.5, and 6.8 [9,10]. It is also known that cationic and dianionic forms occur only at extremely high or low pH [10]. The pKa values differ slightly for different solvents, 3.1, 10.6, and 11.5 for methanol [11] and 0.51, 10.33, and 8.98 for DMSO (dimethyl sulfoxide) [12]. Numerous studies still focus on applications for fluorescein, including in medical bioimaging [13,14,15] or optical sensors [16,17]. Fluorescein derivatives are also used for photonic applications in sol–gel hybrids [18,19,20,21,22] as thin films and SiO_2_/TiO_2_ spheres. This dye group exhibits exceptional photophysical properties, namely of high photostability and fluorescent quantum yield. Additionally, fluorescein preparation is relatively easy and cost-efficient. So far, only limited work has been carried out on its alkylated derivatives. Generally, fluorescein alkylation is accomplished by reaction with an appropriate alkyl halide or dimethyl sulfide in the presence of an inorganic base. Reported yields are rather high and vary from 40% up to even 92% [23,24,25].

The method for synthesis of fluorescent derivative substituted as product A (3′,6′-substituted) and product B (2,6′-substituted) was reported using the same or slightly modified procedures [26,27,28]. The previous literature on the obtained derivatives have shown that it is possible to obtain 2,6′-substituted derivatives with the use of derivatives substituted in the 3′,6′ positions with practically 100% yield [29]. Given this discrepancy, we set out here to carefully investigate the fluorescein alkylation reaction. We were able to successfully modify and simplify the already reported synthesis procedure [30], shortening the reaction time and enhancing the yields of both isolated products.

When considering potential applications and, consequently, designing materials with well-defined emission spectral range, tautomerization and prototropism of fluorescein and its derivatives are particularly problematic phenomena. The pH dependence of fluorescein-based materials prepared by sol–gel synthesis has already been studied for the ring-opened forms (product B) [5,22]. Our paper presents the possibility of doping SiO_2_-based layers with methylated derivative 1B. To obtain such layers, various pH catalysts in a sol–gel silica network synthesis were used, resulting in different emission colors under a UV lamp after gelation, which most likely resulted from the various forms trapped in the matrix.

## 2. Materials and Methods

All the chemical reagents and solvents were obtained from commercial sources and directly used without further purification.

Obtained crude compounds were purified by silica gel chromatography using silica gel 60 (40–63 mesh). TLC (thin layer chromatography) was performed using Macherey-Nagel silica gel Polygram SIL G plates (Macherey-Nagel GmbH & Co., Dueren, Germany).

The results of TLC analyses were checked with UV light (λ = 254 nm). The purity of final compounds (>95%) was confirmed by HPLC (high-performance liquid chromatography) using Varian ProStar 210 (Agilent, Santa Clara, CA, USA) with a dual λ absorbance detector system Discovery^®^ BIO Wide Pore C8 HPLC Column (250 mm × 21.2 mm, 10 μm) (Sigma Aldrich, Darmstadt, Germany) with a 15 mL/min flow rate using a gradient 5−95% (0.05% TFA (trifluoroacetic acid) in acetonitrile) in (0.05% TFA in water) over 15 min (Method A) or Discovery^®^ BIO Wide Pore C8 HPLC Column (250 mm × 4.6 mm, 10 μm) with a 0.9 mL/min flow rate using a gradient 0−100% (0.05% TFA in acetonitrile) in (0.05% TFA in water) over 15 min (Method B) [31].

The nuclear magnetic resonance spectra (NMR) were recorded on a Bruker Avance 600 MHz (600.58 MHz for ^1^H NMR) spectrometer (Bruker, Billerica, MA, USA). Chemical shifts were reported in parts per million (ppm) relative to a tetramethylsilane internal standard. High-resolution mass spectra (HRMS) were acquired on a Waters Acquity Ultra Performance LC, LCT Premier XE (Waters, Milford, MA, USA) [31].

Infrared spectra (FT-IR) were determined using a Tensor 27 FTIR spectrometer (Bruker, Billerica, MA, USA) with an ATR accessory with a diamond crystal in the wavelength range 400–4000 cm^−1^.

The absorption spectra were recorded at room temperature on a Varian Cary 5000 Scan spectrophotometer (Agilent, Santa Clara, CA, USA) in the range 500–400 nm. Measurements of emission and excitation spectra in addition to luminescence quantum yields were recorded at room temperature on an Edinburgh Instruments FLS980 spectrofluorometer (Edinburgh Instruments, Livingston, UK) equipped with a xenon lamp. Optical filters were used during the measurements of photonic layers. The decay time measurements were recorded on an Edinburgh Instruments FLS980 spectrofluorometer equipped with 280 and 360 nm laser diodes.

### 2.1. Synthesis Procedures

#### 2.1.1. General Synthesis Procedure

Fluorescein (1 g, 3 mmol, and 1.0 eq) and appropriate base were dissolved in *N*,*N*-dimethylformamide (DMF) (5 mL). Iodomethane (purity ≥ 99%) was added, and the reaction was continued with vigorous mixing using a magnetic stirrer for 24 h at 70 °C under nitrogen atmosphere. Next, ethyl acetate (30 mL) was added to the reaction mixture followed by washing with 5% citric acid (25 mL). The collected aqueous phases were extracted with ethyl acetate (3 × 25 mL). Finally, the collected organic phases were rinsed with brine and dried over Na_2_SO_4_. The mixture was filtered and evaporated to produce the raw product as an orange solid, which was purified via column chromatography with silica gel as a stationary phase.

#### 2.1.2. General Procedure of Microwave-Assisted Fluorescein Alkylation

Fluorescein (1 g, 3 mmol, 1.0 eq) and cesium carbonate (3.9 g, 12 mmol, 4 eq) were dissolved in DMF (5 mL). The appropriate amount of bromide was added, and the reaction was stirred for 10 min at 70 °C under nitrogen atmosphere in the microwave reactor Milestone. Next, ethyl acetate (30 mL) was added to the resulting mixture followed by washing with 5% citric acid (25 mL). The collected aqueous phases were extracted with ethyl acetate (3 × 25 mL). Finally, the collected organic phases were rinsed with brine and dried over Na_2_SO_4_. The mixture was filtered and evaporated to produce the raw product as an orange solid, which was purified via column chromatography with silica gel as a stationary phase.

#### 2.1.3. General Procedure of Solventless Ball-Milling Fluorescein Alkylation

Fluorescein (1 g, 3 mmol, 1.0 eq) and cesium carbonate (3.9 g, 12 mmol, 4 eq) were placed in a ball mill container made out of agate with two balls (diameter 15 mm). The appropriate bromide was added, and the reaction was carried out for 1 h under nitrogen atmosphere without additional solvent. Upon removal from the mill, the mixture appeared completely homogeneous. Next, the powder mixture was dissolved in ethyl acetate (30 mL) and washed with 5% citric acid (25 mL). The combined aqueous phases were extracted with ethyl acetate (3 × 25 mL). Finally, the combined organic phases were washed with brine and dried over sodium sulfate. The mixture was filtered and evaporated to produce the raw product as an orange solid, which was purified via column chromatography with silica gel as a stationary phase.

#### 2.1.4. General Procedure of the Sol–Gel Synthesis

Eleven milliliters of EtOH (ethanol) (96%) with the dissolved dye were successively introduced into a 25 mL vial to obtain a concentration of 1.5 × 10^−5^ mol in the total volume of the sol, 7 mL of TEOS (tetraethyl orthosilicate) (purity ≥ 99%, Sigma Aldrich, Steinheim, Germany), 1 mL of 0.1 M HCl (obtained by diluting with 35% HCl analytical grade), or 1 mL of 0.1 M NaOH (solution prepared from NaOH granules dissolved in distilled water). The vial was covered with parafilm and ultrasonicated for 3 h in a water bath at a temperature 50 °C. Next, the sol was placed in several glass vials and allowed to gel in a fume cupboard for about 2 weeks. Materials obtained in this way were subjected to further spectroscopic characterization.

## 3. Results and Discussion

**Figure 1:**
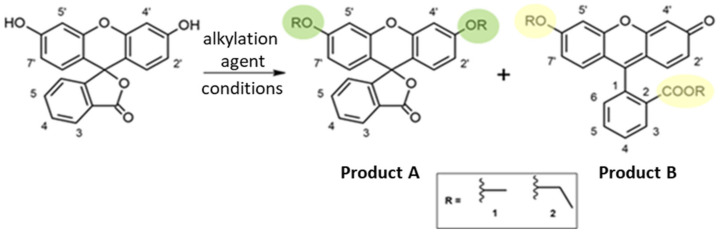
Reaction scheme. The positions 3′ and 6′ (products A, closed forms) are marked in green and the positions 2 and 6′ (products B, open forms) are marked in yellow. Later in the text, 1A/1B are methylated, while 2A/2B are ethylated derivatives.

We present the results for the synthesis of two alkylated derivatives—closed and open forms (shown in Figure 1)—with critical reference to the methods described so far in the literature [26,27,28,29]. Compared to another synthesis study [29], we managed to obtain both a di-*O*-alkylated derivative A and an ether–ester derivative B using fluorescein. Yields of prepared fluorescein derivatives are listed in Table 1. It should be noted that in work describing the simultaneous synthesis of two derivatives, the lactone form was used for the synthesis of the ether–ester derivative [32] and not fluorescein, as in the case in our study.

### 3.1. Synthesis Optimization

**Figure 2:**
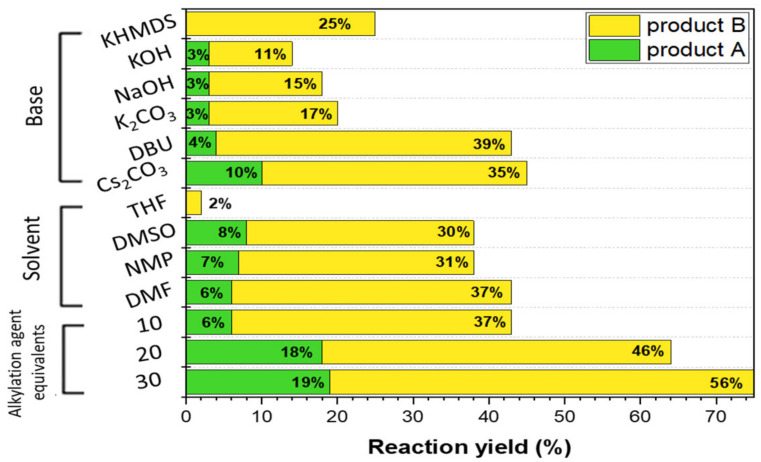
Selection of reaction *O*-alkylation parameters for the classical heating procedure (KHMDS—potassium *bis*(trimethylsilyl)amide; DBU—1,8-diazabicyclo(5.4.0)undec-7-ene; THF—tetrahydrofuran; DMSO—dimethyl sulfoxide; NMP—*N*-methyl-2-pyrrolidone; and DMF—dimethylformamide). More details in Section 3.1 Synthesis Optimization.

We investigated the influence of the solvent, base, and methyl iodide (alkylation agent) equivalents, aiming to obtain the best product A to product B ratio and highest possible reaction yield. The ratio of products closest to 1:1 allows efficient isolation of the largest possible amounts of both derivatives. By selecting optimal reaction conditions (cesium carbonate as base, DMF, and 30 eq of methyl iodide) an improvement in the product A:B ratio (from 1:6 to 1:3) was achieved with the highest possible yield (75%). The findings of our investigations are summarized in Figure 2. In the first step, our goal was to select the best base using the same amount of alkylation agent equivalents (10 eq) and the same solvent (DMF), which turned out to be cesium carbonate. Next, reactions were carried out in various solvents with the same quantity of Cs_2_CO_3_ and alkylation agent. DMF was confirmed as the best solvent. The final optimization step was to determine the amount of alkylating agent (methyl iodide), with the best results for 30 eq.

Now that the optimal conditions had been established, the next step of our research focused on microwave acceleration of the reaction. To the best of our knowledge, this is the first time that both alkyl fluorescein derivatives (products A and B) have been prepared using a microwave reactor. As we anticipated, the use of microwave instead of the classical heating of the reaction mixture shortened the reaction time, enhanced the reaction yield, and increased the purity of the products [33,34]. Other studies on the synthesis of fluorescein alkylated derivatives required longer reaction times (several hours up to 24 h [30,35,36,37]) or used more than one synthesis step [23] in comparison to our synthesis method.

Our procedure allowed us to significantly shorten the reaction time from 24 h to only 10 min and simplify the synthesis procedure. It is also worth noting that the overall reaction efficiency improved for different alkylating agents—to 97% and 87% for reactions with methyl iodide and ethyl bromide, respectively. However, in both cases—the classic heating and microwave conditions—the main product was an ether–ester derivative (product B). We also found that the elongation of the alkyl halide chain did not significantly affect either the product ratio or the reaction efficiency.

### 3.2. New Route—Ball Milling

**Figure 3:**
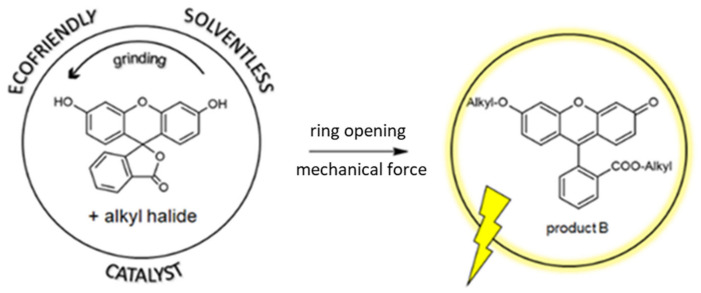
Schematic diagram for a ball-milling reaction.

**Figure 4:**
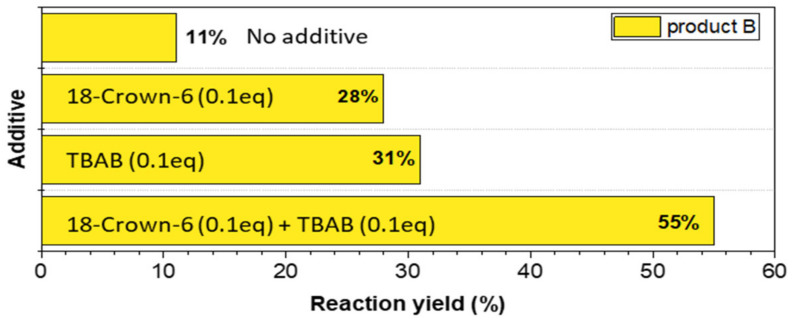
Optimization of solventless ball-milling fluorescein alkylation (TBAB—tetrabutylammonium bromide).

Fluorescein alkylation was also carried out using a ball mill as a potential reaction reactor (schematic diagram shown in Figure 3). Many studies on using a ball mill for organic synthesis have been published, emphasizing the economic and ecological aspects [38,39]. It was proven to be an efficient promoter of the oxidative ring-opening reaction [40] in different dye families. As reported in the literature [41] the addition of a phase transfer catalyst significantly improved the methylation yield. The optimization of the process is presented in Figure 4. Owing to the low reaction yields without the use of the catalyst, 18-crown-6 ether and tetrabutylammonium bromide (TBAB) were used in the subsequent optimization steps, the addition of which significantly improved the methylation yield to 28% and 31%, respectively. In our research, the best result (55% yield) was obtained for 0.1 eq 18-crown-6 and 0.1 eq TBAB used simultaneously. Comparing the obtained results for the reactions using classical heating and ball milling, we noticed practically similar yields for both methyl and ethyl 2,6′ derivatives. However, using the ball mill allows only one fluorescent product B to be obtained because of the mechanical force, which indicates the high selectivity of the reaction. The solvent-free nature and use of the most selective catalysts in the developed method are in perfect alignment with the green chemistry trend. By eliminating the use of harmful solvents, the developed method is friendlier to health and the environment. Another advantage of this method is its efficiency, short duration, and lower amounts of byproduct formation, which obviously reduce pollution and facilitate isolation of the final compound. Another undeniable advantage of this solventless method is the reduction in reaction time and high yields of the obtained product. Thus, the method can be applied for the fast and efficient synthesis of ether–ester derivatives of fluorescein with longer alkyl chains.

### 3.3. Spectroscopic Properties

Fluorescein luminescence is strongly dependent on solvent pH, and changes in pH are visible in the shape of emission spectra, quantum yield values, and decay times [4,5,7,42]. Fluorescein occurs in several prototropic forms—cationic, neutral, and anionic (including monoanion and dianion), but because of its superior quantum efficiency, is mostly used in dianionic form [43,44]. Our goal was to first examine their spectra and then to incorporate different prototropic fluorescein forms into the matrix.

**Figure 5:**
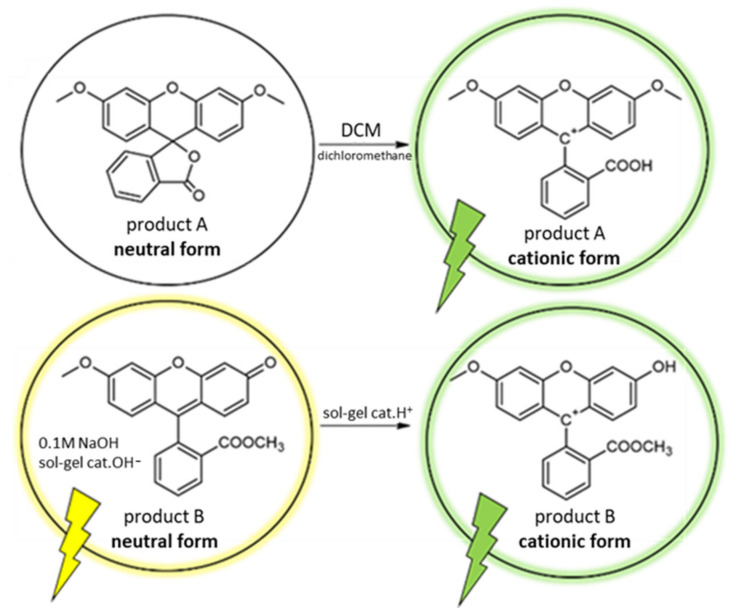
Proposed structures of prototropic forms and their occurrence based on spectroscopic analysis.

For this, the spectroscopic properties of fluorescein solutions in different solvents were immediately measured upon dissolution. The absorption and emission spectra were recorded for respective 1.0 × 10^−5^ M solutions of product B in 0.1 M NaOH and product A in dichloromethane (DCM). Different solvents were used because, despite literature reports, we encountered difficulties in dissolving product A in 0.1 M NaOH, owing to the rigid structure of methylated and ethylated derivatives containing the lactone ring mentioned in the literature [45]. Moreover, in contrast to the original dye, the derivatives may take less-pH-dependent forms. The observed derivatives are mentioned in Figure 5. Decay times were obtained by laser diode excitation.

Based on NMR studies (described in the Supplementary Materials (Section S1) on the structures of synthesized neutral forms, we proposed chemical formulas of the obtained prototropic forms shown in Figure 5. Since the closed neutral form (product A) was colorless and non-fluorescent, we took advantage of the fact that the DCM used is an aprotic solvent, which favors lactone ring opening in different dye groups [46]. Therefore, we expected to observe the cationic form even at neutral pH value. The structure of product B, on the other hand, did not allow for the formation of a monoanion or dianion form. This was crucial, since the UV–Vis absorption and emission spectra of the neutral form were similar to the monoanion one (owing to little participation of the carboxyl group in ionization [7]).

A comparison of the results we obtained with previously published spectroscopic data for parent fluorescein prototropic forms is summarized in Table 2. It should be emphasized that the obtained results may differ, not because the fluorescein itself was tested but because of its modified substituted derivatives. In both cases, the absorption/emission (shown in the Supplementary Materials Figures S2.1 and S2.2)) spectra were analogous to derivatives 1 and 2. In the case of QY, our measurements differed visibly from those in the literature reports because different techniques were used. In our study, quantum yield was determined by using an integrating sphere, while in the literature, it was obtained from φ_f_ = τ_f_/τ_r_, where τ_r_ is the radiative lifetime [47].

### 3.4. Sol–Gel Hybrid Materials

**Figure 6:**
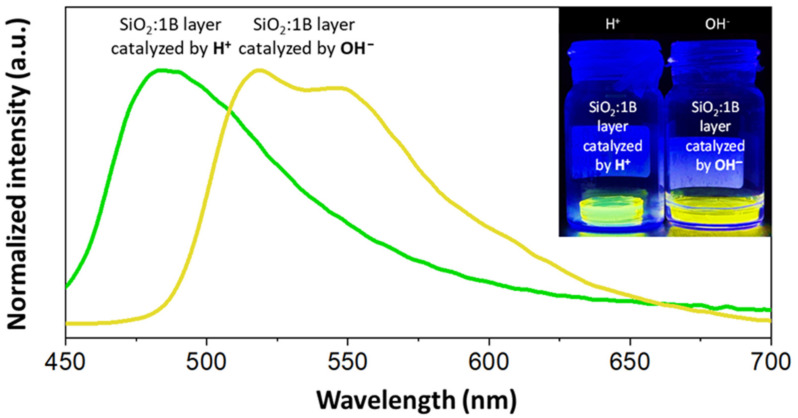
Emission spectra for SiO_2_ gel layers doped with derivative 1B (λ_ex_ for H^+^ catalyzed material = 320 nm, while for OH^−^ catalyzed = 360 nm); inset: photograph of dried gels under UV light (λ_ex_ = 365 nm).

When considering the trapping attempts of different forms of the same dye in the matrix, different basic and acidic catalysts were used to obtain different prototropic forms. Methylated open forms were added into the sol–gel reaction in ethanolic solution. Dried gels showed bright emission under UV light excitation, as presented in Figure 6, as well as emission spectra, and absorption spectra are shown in the Supplementary Materials (Figures S2.1–S2.3). Measured emission maxima for H^+^ catalyzed layers (λ_ex_ = 320 nm) were placed at 480 nm, while for OH^−^ they were at 517 nm. The first spectrum extended to 140 nm, and the FWHM (full width at half maximum) was approximately 75 nm. We also measured the spectra after 90 days to test the stability of the obtained materials (comparison is shown in the Supplementary Materials (Figure S2.4)). Spectroscopic properties remained comparable to those presented in Figure 6, which pointed to the stability of the sol–gel layers.

**Figure 7:**
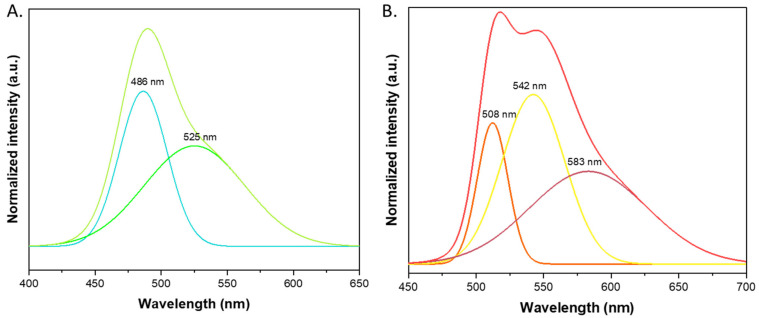
Gaussian band deconvolution of emission spectra for SiO_2_ gel layers doped with derivative 1B. (**A**). Catalyzed by H^+^, (**B**). Catalyzed by OH^−^.

Since there were shoulders and overlapping visible spectra, we attempted to deconvolute the emission spectra into Gaussian shape bands, presented in Figure 7. The shape of the bands may be caused by ring stretching. Deconvolution (presented in Figure 7, tables in Supplementary Materials Tables S2.1. and S2.2.) was carried out with Origin (version 2021, OriginLab, Northampton, MA, USA) software using analyzing options. For the H^+^ catalyzed layer, we showed two main peaks with maxima at 485 nm (FWHM = 36 nm; 1866 cm^−1^) and 525 nm (FWHM = 76 nm; 2761 cm^−1^), responsible for the wide shoulder observed in the spectrum. The visible difference in the position of the maximum emission of the cationic forms (DCM solution of product A and layers catalyzed by H^+^) may result in the different form structures and matrix occurrences. The second spectrum most likely consisted of three components with FWHM values of 23, 46, and 87 nm (880, 1980, and 2853 cm^−1^), respectively. The dye trapped in the SiO_2_ was probably present in cationic form for the H^+^- and neutral for the OH^−^-catalyzed layer, respectively.

## 4. Conclusions

To conclude, we tested three different methods for fluorescein alkylation using various reaction conditions. Our research showed the possibility of simultaneously obtaining stable derivatives substituted in different positions with the appropriate alkyl chains as a result of both conventional and microwave heating. This work allowed finding efficient fast ways to synthesize both closed and open forms simultaneously. In addition, a microwave-assisted route for synthesis was developed. Using a microwave reactor shortened the reaction time to 10 min while maintaining high yields, which is an undoubted advantage. The ball mill is clearly an eco-friendly method that facilitates product isolation; however, it is not possible to obtain two derivatives simultaneously. Spectroscopic data were presented for compound B in 0.1 M NaOH and for A in DCM. Taking into consideration the structure of the obtained products, neutral and cationic forms were observed. Additionally, SiO_2_ layers doped with 1B were prepared with catalyzation with H^+^/OH^−^. The main advantage of this synthesis technique is that it allows trapping both the cationic and the neutral form derived from the same dye in a silica network, resulting in emissions in different spectral regions. Using different amounts of sol allows for the synthesis of layers of different thicknesses. Compared to previous literature reports, we were able to obtain materials with different emission colors from the same starting organic dye.

## Figures and Tables

**Table 1:** Reaction yields of prepared alkylated derivatives.

Entry	Microwave Heating Yield (%)		Classical Heating Yield (%)		Ball-Milling Yield (%)
	A	B	A	B	B
1 (methylated derivatives)	29	68	19	56	55
2 (ethylated derivatives)	25	62	15	35	41

**Table 2:** Spectroscopic properties of products B and A and parent fluorescein prototropic forms and comparison with the literature.

Origin	Compound	λ_abs_ (nm)	λ_em_ (nm)	T (nm)	QY (%)	Concluded Form
Reference	Fluorescein	437	475	3.5–4.4	90–100	Cationic [4,7,42,47]
	Fluorescein	453; 474	517	2.97 ± 0.02	29	Neutral [4,7,42,47]
This work	1B (methylated open form)	455; 475	515	3.23	37.88	Neutral
	2B (ethylated open form)	452; 473	517	3.24	36.56	Neutral
	1A (methylated closed form)	440	460	4.28	23.18	Cationic
	2A (ethylated closed form)	440	460	3.49	19.89	Cationic

## Data Availability

Data are contained within the supplementary material.

## Supplementary Materials

The following are available online at https://www.mdpi.com/article/10.3390/ma15010203/s1. Section S1: General Characterization of Obtained Derivatives. Section S2: Spectroscopic Properties. Figure S2.1. Emission and absorption spectra measured for (a) product 1A in DCM; (b) product 1B in 0.1M NaOH. Figure S2.2. Emission and absorption spectra measured (a) product 2A in DCM; (b) product 2B in 0.1M NaOH. Figure S2.3. Gels’ absorption spectra. Figure S2.4. Gels’ emission spectra measured in comparison after 90 days. Table S2.1. Deconvolution table for SiO_2_ gel-layers doped with derivative 1B catalyzed by H^+^. Table S2.2. Deconvolution table for SiO_2_ gel-layers doped with derivative 1B catalyzed by OH^−^.
