# Supplementary material for: Development of Efficient One-Pot Methods for the Synthesis of Luminescent Dyes and Sol–Gel Hybrid Materials
Source: Materials (Basel). 2021 Dec 28;15(1):203. doi: 10.3390/ma15010203 (PMC8746091; doi:10.3390/ma15010203)
Supplement: Supplementary file 1 [file materials-15-00203-s001.zip › materials-1473764-SM.pdf]

Supplementary Materials

Development of Efficient One-Pot Methods for the Synthesis of Luminescent Dyes and Sol–Gel Hybrid Materials

Maria Zdończyk [1,2], Bartłomiej Potaniec [2], Marcin Skoreński [2] and Joanna Cybińska [1,2,*]

[1] Faculty of Chemistry, University of Wroclaw, F. Joliot-Curie 14 Street, 50-383 Wrocław, Poland; maria.zdonczyk@chem.uni.wroc.pl (M.Z.)

[2] Advanced Materials Synthesis Group, Łukasiewicz Research Network—PORT Polish Center for Technology, Stabłowicka 147 Street, 54-066 Wrocław, Poland; bartlomiej.potaniec@port.lukasiewicz.gov.pl (B.P.); marcin.skorenski@port.lukasiewicz.gov.pl (M.S.)

* Correspondence: joanna.cybinska@chem.uni.wroc.pl or joanna.cybinska@port.lukasiewicz.gov.pl

S1. General Characterization of Obtained Derivatives

3′,6′-Dimethoxy-3H-spiro[isobenzofuran-1,9′-xanthen]-3-one (1- product A)

^{1}H NMR (500 MHz, DMSO-*d6*) δ 8.03 (dd, *J* = 7.6, 0.8 Hz, 1H), 7.80 (td, *J* = 7.5, 1.1 Hz, 1H), 7.74 (td, *J* = 7.5, 0.9 Hz, 1H), 7.28 (d, *J* = 7.6 Hz, 1H), 6.93 (d, *J* = 2.5 Hz, 2H), 6.75 (d, *J* = 2.5 Hz, 1H), 6.73 (d, *J* = 2.5 Hz, 1H), 6.71 – 6.67 (m, 2H), 3.83 (s, 6H); **FTIR-ATR** [cm^{-1}]: 3052.78. 3006.32, 2917.19, 2832.99, 1761.4, 1632.01, 1613.42, 1576.22, 1502.01, 1464.45, 1435.28, 1421.05, 1350.67, 1283.07, 1247.29, 1207.56, 1170.46, 1161.16, 1108.89, 1097.53, 1079.93, 1029.26, 988.75, 956.93, 879.93, 827.79, 794.20, 766.36, 735.27, 694.57, 669.95, 650.68, 621.11, 610.39, 587.82, 531.67, 485.02, 466.31, 450.96, 433.85; **LC-MS**: t_R= 20.1 min; **MS**: calcd for $(C_{22}H_{16}O_5)H^+$, 361.1; found 361.2.

Methyl 2-(3-hydroxy-6-methoxy-3H-xanthen-9-yl)benzoate (1- product B)

^{1}H NMR (500 MHz, DMSO-*d6*) δ 8.22 (dd, *J* = 7.9, 1.0 Hz, 1H), 7.88 (td, *J* = 7.5, 1.3 Hz, 1H), 7.79 (td, *J* = 7.7, 1.3 Hz, 1H), 7.50 (dd, *J* = 7.6, 0.9 Hz, 1H), 7.23 (d, *J* = 2.4 Hz, 1H), 6.90 (dd, *J* = 8.9, 2.4 Hz, 1H), 6.85 (d, *J* = 8.9 Hz, 1H), 6.81 (d, *J* = 9.7 Hz, 1H), 6.39 (dd, *J* = 9.7, 1.9 Hz, 1H), 6.25 (d, *J* = 1.9 Hz, 1H), 3.92 (s, 3H), 3.59 (s, 3H); **FTIR-ATR** [cm^{-1}]: 3606.27, 3377.38, 3001.61, 2947.29, 2846.54, 1724.52, 1642.90, 1586.78, 1541.09, 1506.36, 1479.35, 1452.11, 1414.65, 1378.30, 1345.79, 1272.68, 1255.33, 1210.40, 1139.10, 1104.42, 1078.05, 1023.73, 965.51, 932.15, 917.79,898.05, 851.57, 822.70, 797.45, 750.58, 714.61, 705.43, 662.69, 624.53, 600.52, 589.69, 569.21, 527.04, 486.80,443.18; **LC-MS**: t_R= 15.2 min; **MS**: calcd for $(C_{22}H_{16}O_5)H^+$, 361.1; found 361.2.

3′,6′-diethoxy-3H-spiro[isobenzofuran-1,9′-xanthen]-3-one (2- product A)

^{1}H NMR (500 MHz, DMSO-*d6*) δ 8.01 (dd, *J* = 7.6, 0.9 Hz, 1H), 7.79 (td, *J* = 7.5, 1.1 Hz, 1H), 7.73 (td, *J* = 7.5, 0.9 Hz, 1H), 7.27 (d, *J* = 7.6 Hz, 1H), 6.89 (d, *J* = 2.4 Hz, 1H), 6.72 (d, *J* = 2.5 Hz, 1H), 6.70 (d, *J* = 2.5 Hz, 1H), 6.67 (s, 1H), 6.65 (s, 1H), 4.09 (q, *J* = 7.0 Hz, 4H), 1.33 (t, *J* = 7.0 Hz, 6H); **FTIR-ATR** [cm^{-1}]: 2982.10, 2921.70, 2850.78, 1747.76, 1616.12, 1600.13, 1569.11, 1504.73, 1464.19, 1427.74, 1394.12, 1355.10, 1327.18, 1284.81, 1272.40, 1247.93, 1224.11, 1191.66, 1163.46, 1104.66, 1082.97, 1042.68, 1012.13, 1001.14, 968.66, 950.32, 936.92, 898.03, 869.42, 837.70, 824.76, 816.17, 785.34, 759.29, 694.81, 671.40, 650.87, 638.69, 616.63, 583.63, 521.28, 510.87, 463.72, 450.28,417.92; **LC-MS**: t_R= 15.2 min; **MS**: calcd for $(C_{24}H_{20}O_5)H^+$, 389.1; found 389.3.

ethyl 2-(6-ethoxy-3-oxo-3H-xanthen-9-yl)benzoate (2- product B)

^{1}H NMR (500 MHz, DMSO-*d6*) δ 8.19 (dd, *J* = 7.9, 1.1 Hz, 1H), 7.86 (td, *J* = 7.5, 1.3 Hz, 1H), 7.78 (td, *J* = 7.7, 1.3 Hz, 1H), 7.50 (dd, *J* = 7.6, 1.0 Hz, 1H), 7.20 (d, *J* = 2.3 Hz, 1H), 6.91 – 6.79 (m, 3H), 6.39 (dd, *J* = 9.7, 1.9 Hz, 1H), 6.23 (d, *J* = 1.9 Hz, 1H), 4.19 (q, *J* = 7.0 Hz, 2H), 4.02 – 3.89 (m, 2H), 1.36 (t, *J* = 7.0 Hz, 3H), 0.86 (t, *J* = 7.1 Hz, 3H); **FTIR-ATR** [cm^{-1}]: 3388.66, 3059.18, 2981.42, 2938.35, 1718.91, 1643.24, 1588.98, 1540.65, 1504.77, 1468.38, 1452.27,

1413.96, 1400.70, 1377.56, 1344.98, 1253.23, 1208.74, 1167.41, 1139.58, 1103.50, 1079.44, 1041.33, 986.00, 959.98, 921.84, 878.96, 851.75, 822.41, 754.96, 709.74, 661.08, 621.69, 612.16, 589.79, 570.59, 495.40, 462.55, 439.86, 416.44; **LC-MS:** t_R= 21.1 min; **MS**: calcd for $(C_{24}H_{20}O_5)H^+$, 389.1; found 389.3.

S2. Spectroscopic Properties

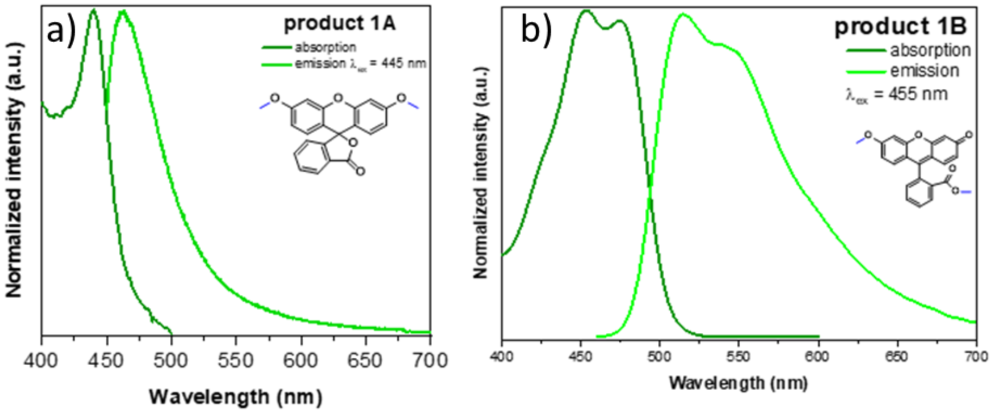

Figure S2.1. Emission and absorption spectra measured for: (**a**) product 1A in DCM; (**b**) product 1B in 0.1M NaOH.

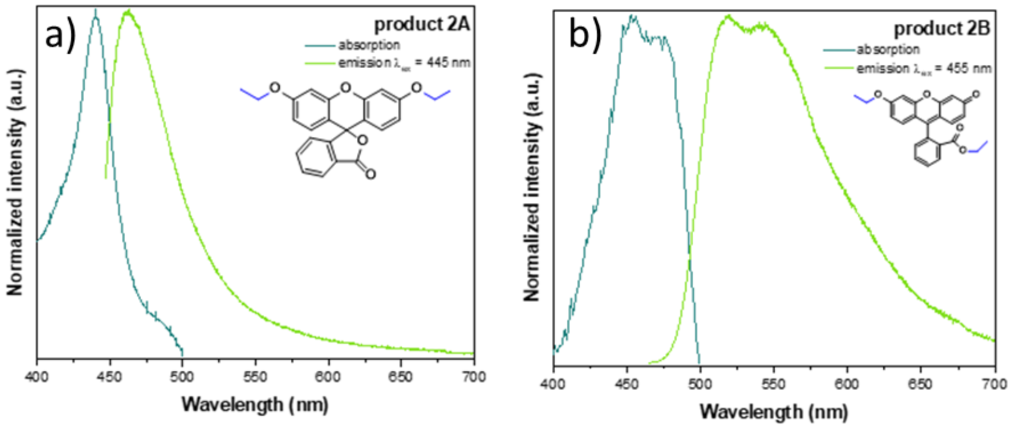

Figure S2. 2. Emission and absorption spectra measured: (**a**) product 2A in DCM; (**b**) product 2B in 0.1M NaOH.

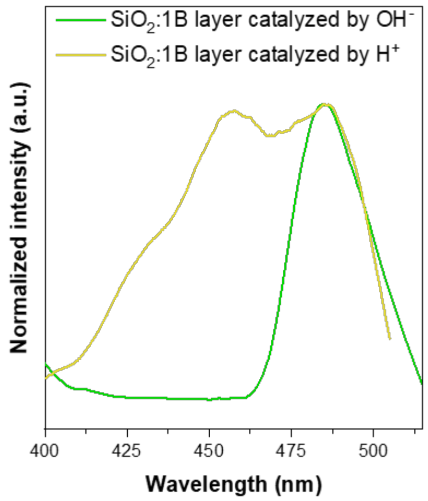

Figure S2.3. Gels' absorption spectra.

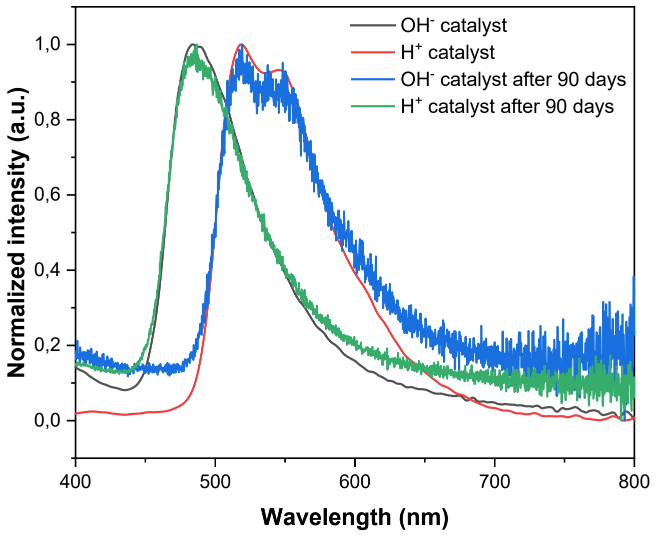

Figure S2.4. Gels' emission spectra measured in comparison after 90 days.

Table S2.1. Deconvolution table for SiO_2 gel-layers doped with derivative 1B catalyzed by H^+.

**Model**	**Gauss**	
Equation	$$y = y_0 + \frac{A}{\left(w \times \sqrt{\frac{\Pi}{2}}\right) \times e^{\left(\frac{-2(x-x_c)}{w}\right)^2}}$$	
Plot	Peak 1	Peak 2
y_0	0.09908 ± 0.00124	0.09908 ± 0.00124
x_c	486.41472 ± 0.15742 nm	525.15791 ± 1.30576 nm
w	36.29697 ± 0.58509 nm	76.07913 ± 1.35126 nm
FWHM	1866 cm^{-1}	2761 cm^{-1}
A	29.75164 ± 1.15786	40.41715 ± 1.35372
Reduced Chi-Sqr	0.00109	
R-Square (COD)	0.98492	
Adj. R-Square	0.98486	

Table S2. 2. Deconvolution table for SiO_2 gel-layers doped with derivative 1B catalyzed by OH^-.

**Model**	**Gauss**		
Equation	$y = y_0 + \frac{A}{\left(w \times \sqrt{\frac{\Pi}{2}}\right) \times e^{(\frac{-2(x-x_c)}{w})^2}}$		
Plot	Peak 1	Peak 2	Peak 3
y_0	$0.03505 \pm 3.22203{\cdot}10^{-4}$	$0.03505 \pm 3.22203\ 10^{-4}$	$0.03505 \pm 3.22203{\cdot}10^{-4}$
x_c	512.23321 ± 0.04897 nm	542.4366 ± 0.21751 nm	583.25472 ± 0.75272 nm
w	22.74157 ± 0.15506 nm	46.44193 ± 0.50766 nm	87.30921 ± 0.59525 nm
FWHM	880 cm^{-1}	1980 cm^{-1}	2853 cm^{-1}
A	15.12764 ± 0.29947	37.07062 ± 0.86908	38.11507 ± 0.69258
Reduced Chi-Sqr	$5.43755{\cdot}10^{-5}$		
R-Square (COD)	0.99945		
Adj. R-Square	0.99945		
